# Magnetization Reversal Process and Magnetostatic Interactions in Fe_56_Co_44_/SiO_2_/Fe_3_O_4_ Core/Shell Ferromagnetic Nanowires with Non-Magnetic Interlayer

**DOI:** 10.3390/nano11092282

**Published:** 2021-09-02

**Authors:** Javier García, Alejandro M. Manterola, Miguel Méndez, Jose Angel Fernández-Roldán, Víctor Vega, Silvia González, Víctor M. Prida

**Affiliations:** 1 Departamento de Física, Facultad de Ciencias, Universidad de Oviedo, C/Federico García Lorca No. 18, 33007 Oviedo, Spain; alevale71@gmail.com (A.M.M.); miguel.mendez82@gmail.com (M.M.); fernandezroljose@uniovi.es (J.A.F.-R.); gonzalezgana@uniovi.es (S.G.); 2 Laboratorio de Membranas Nanoporosas, Edificio de Servicios Científico Técnicos “Severo Ochoa”, Universidad de Oviedo, C/Fernando Bonguera s/n, 33006 Oviedo, Spain; vegavictor@uniovi.es

**Keywords:** nanoporous anodic alumina template, electrodeposition, ALD, magnetic nanowire and nanotube, core/shell nanostructure, FORC analysis, MOKE, magnetization reversal, spintronics

## Abstract

Nowadays, numerous works regarding nanowires or nanotubes are being published, studying different combinations of materials or geometries with single or multiple layers. However, works, where both nanotube and nanowires are forming complex structures, are scarcer due to the underlying difficulties that their fabrication and characterization entail. Among the specific applications for these nanostructures that can be used in sensing or high-density magnetic data storage devices, there are the fields of photonics or spintronics. To achieve further improvements in these research fields, a complete understanding of the magnetic properties exhibited by these nanostructures is needed, including their magnetization reversal processes and control of the magnetic domain walls. In order to gain a deeper insight into this topic, complex systems are being fabricated by altering their dimensions or composition. In this work, a successful process flow for the additive fabrication of core/shell nanowires arrays is developed. The core/shell nanostructures fabricated here consist of a magnetic nanowire nucleus (Fe_56_Co_44_), grown by electrodeposition and coated by a non-magnetic SiO_2_ layer coaxially surrounded by a magnetic Fe_3_O_4_ nanotubular coating both fabricated by means of the Atomic Layer Deposition (ALD) technique. Moreover, the magnetization reversal processes of these coaxial nanostructures and the magnetostatic interactions between the two magnetic components are investigated by means of standard magnetometry and First Order Reversal Curve methodology. From this study, a two-step magnetization reversal of the core/shell bimagnetic nanostructure is inferred, which is also corroborated by the hysteresis loops of individual core/shell nanostructures measured by Kerr effect-based magnetometer.

## 1. Introduction

Cylindrical coaxial nanostructures can exhibit novel phenomena due to their unique size- and shape-dependent physico-chemical properties, as compared to their bulk counterparts, making them enormously attractive as innovative multifunctional materials, for both fundamental and technological applications, such as photonics, drug delivery, and cell separation for proteomics research, hierarchical core/shell heterostructured electrodes for high-performance Li-ion batteries, or highly anisotropic ferromagnetic-antiferromagnetic core/shell nanomaterials exhibiting tunable magnetic properties [1,2,3,4]. These peculiar, elongated hybrid nanostructures are coaxially combining two or more components with several phases of distinct properties that could originate additional effects compared with single-phase nanomaterials, which outfit them with enhanced multifunctional properties. For example, core/shell ferromagnetic nanowires (NWs) and nanotubes (NTs) comprising hybrid metallic/non-metallic core and shell materials usually offer the specific advantages of mixed properties by combining both contributions to convey with tailored multifunctional capabilities, such as the high magnetization and magnetic anisotropy values present in transition metals (TM = Fe, Ni, Co, and their alloys), with the outstanding biocompatibility and enhanced chemical stability exhibited by oxide layers (SiO_2_, TiO_2_, Fe_3_O_4_, NiO, CoO, etc.) [5,6,7,8].

Several methods have been successfully developed up to date to synthesize these cylindrical coaxial hetero-nanostructures. Among them, the low-cost template-assisted method can be easily combined with other techniques for the fabrication of hybrid core/shell functional nanomaterials, such as electrochemical deposition [9,10], wet-chemical route and coprecipitation [11,12], atomic layer deposition (ALD) [13,14,15,16,17], and chemical vapor deposition (CVD) [18]. For this reason, as well as for its high reproducibility and simplicity, this additive fabrication strategy constitutes one of the most widespread methods for the synthesis of one- and two-dimensional (1D and 2D) coaxial nanostructures. At the same time, three-dimensional (3D) core/shell magnetic nanostructures are nowadays appealing for novel applications in magnetic recording, biotechnology, nanoelectronics, plasmonics, and spintronics devices [6,19,20,21,22]. Among the vast variety of magnetic heterostructures, bimagnetic core/shell nanocomposites, where both the core and shell couple magnetically hard and soft phases, have recently increased their significance due to the novel physical phenomena they can exhibit. These bimagnetic core/shell nanostructures could efficiently tune their magnetic properties such as the thermal stability of saturation magnetization, high magnetocrystalline anisotropy, and coercivity, through the control of the core/shell parameters, including their shape, size, and chemical composition, showing peculiar and unique features such as large exchange bias, tailored blocking temperatures, tunable coercivities, and stepwise switching of magnetization reversal process [23]. The peculiar features emerging from geometrically curved magnetic nanostructures, including 2D nanowires or 3D curved shells and nanotubes, are strongly related to the size- and shape-dependent effects driven by the interplay between the nanomaterial geometry and topology of each magnetic sub-system [24]. The broad range of emergent physical properties makes these three-dimensional curved nanostructures appealing in view of fundamental research on, e.g., skyrmionic systems, photonic and magnonic crystals, or spintronics architectures, also exhibiting these 3D shaped nanomaterials potential applications in photonic, plasmonic, and for energy-efficient racetrack magnetic memory devices [25].

Different types of ferromagnetic biphasic nanomaterials have already been reported, including soft/hard and hard/soft core/shell bimagnetic nanostructures, respectively [26,27]. However, few works report on three-layered core/shell cylindrical NWs systems recently investigated, where the magnetization switching processes in these hybrid nanostructures remain yet unexplored [13,28,29,30,31,32,33].

In this study, we investigate the switching process of ferromagnetic/non-magnetic/ferrimagnetic core/shell nanocomposite made of Fe_56_Co_44_(core)/SiO_2_(interlayer)/Fe_3_O_4_(shell). This hybrid core/shell nanocomposite was experimentally synthesized by a template-assisted additive fabrication strategy based on nanoporous alumina membranes. The external NT shell of magnetite and the subsequent non-magnetic SiO_2_ thin interlayer were deposited by atomic layer deposition (ALD), coupled with the electrochemical deposition technique in order to grow the inner NW core of metallic FeCo alloy. Magneto-optical Kerr effect (MOKE) measurements allow to access the switching process of individual NWs and core/shell nanostructures in order to determine the switching of each layer, either the external shell or the inner core of the whole wire/tube nanomaterial. Moreover, the magnetic properties exhibited by these core/shell nanostructures as well as the magnetostatic interactions between the two magnetic contributions of the bimagnetic nanocomposite were investigated by vibrating sample magnetometry (VSM). However, due to the complexity of the system, the core/shell nanowires have to be characterized while they are still embedded in the matrix array. For this purpose, analysis of magnetization reversal curves by means of First Order Reversal Curves (FORC) was also performed for the FeCo NW core, the shell magnetite NT, and the core/shell NW/NT, respectively, with the aim of complementing hysteretic studies regarding the magnetostatic dipolar interaction of the systems, thus allowing us to extract the individual magnetic properties of each nanostructure from the global magnetic behavior of the samples. Our results established that the magnetization of the FeCo core and the magnetite shell may switch at different switching field values during the magnetization reversal of the core/shell NW/NT nanostructure. This confirms the reversal mechanism of our experimental core/shell NWs is uniquely promoted through magnetostatic dipolar coupling between the NT and NW ferromagnetic layers, because of the direct exchange decoupling between both magnetic layers. These results set an appealing strategy for the design of novel 3D magnetic storage and spintronic devices based on core/shell cylindrical NWs that has not been clearly envisaged up to now.

## 2. Materials and Methods

**Figure 1:**
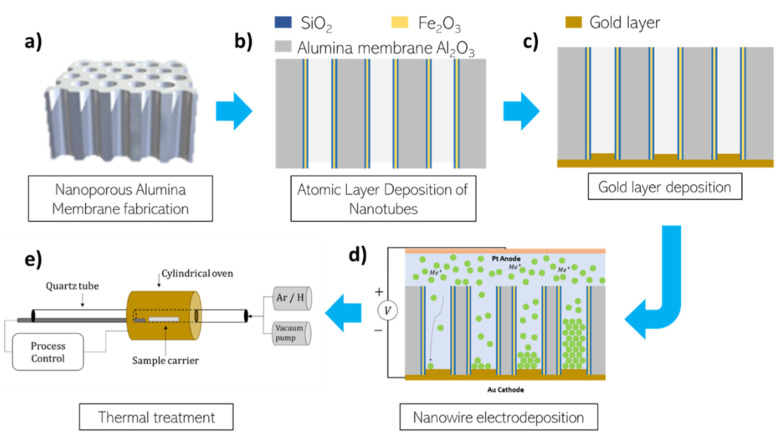
Scheme of the additive fabrication process process to obtain SiO_2_/Fe_2_O_3_/SiO_2_/FeCo core/shell nanotubes/nanowires, and further thermal treatment for Fe_2_O_3_ (hematite) reduction into Fe_3_O_4_ (magnetite). (**a**) Nanoporous alumina template, (**b**) deposition of nanotubes shell by ALD, (**c**) gold contact layer deposition by sputtering, (**d**) electrochemical deposition of metallic nanowire core, (**e**) thermal annealing under reducing atmosphere.

In this section, all steps for FeCo/Fe_3_O_4_ core/shell nanowires fabrication are explained in detail. The complete process flow followed for sample preparation is summarized in Figure 1. First, anodized alumina membranes were fabricated and used as templates for further steps (Figure 1a). The process continues with deposition of the trilayer SiO_2_/Fe_2_O_3_/SiO_2_ by means of atomic layer deposition, thus forming nanotubes inside the pores of the alumina membranes employed as templates (Figure 1b). After the deposition of a sputtered (and thickened by electrodeposition) Au seed layer on one side of the membrane (Figure 1c), Fe_56_Co_44_ nanowires were electrodeposited inside the former NTs, constituting then the magnetic core of the core/shell nanostructure (Figure 1d). Once the core/shell structure is formed, it is necessary to perform a further thermal treatment to reduce the external layer made of Fe_2_O_3_ (hematite) into Fe_3_O_4_ (magnetite), therefore, the shell becomes ferrimagnetic (Figure 1e).

### 2.1. Nanoporous Alumina Membrane Template Fabrication

Hard-Anodized Nanoporous Alumina Membranes (HA-NAMs) were fabricated from electropolished (perchloric acid in ethanol at a rate of 25:75 under an applied voltage of 20 V) high purity aluminum foils (Al 99.999%) using the known process of hard anodization [34,35,36]. The Al substrates were anodized using 0.3 M oxalic acid (C_2_H_2_O_4_) at 4 °C for 1.5 h, under an applied voltage of 140 V. After this process, the remaining aluminum substrate was removed using a solution of 36 g/L of CuCl_2_·2H_2_O and 500 mL/L of HCl at 37%. As a final step, the alumina membrane undergoes a process of wet chemical etching to open the barrier layer of the pores using 5% wt. phosphoric acid (H_3_PO_4_) at 30 °C for 2.5 h leading to a mean pore diameter of 180 nm and a mean interpore distance of 300 nm.

### 2.2. Fabrication of the Nanotubes Shell by Atomic Layer Deposition

The shell of the nanowires, consisting of a three-layered structure is composed of an external layer of silicon dioxide (SiO_2_), a central layer of iron oxide (III) (Fe_2_O_3_), and an internal layer of SiO_2_, which were grown using the Atomic Layer Deposition (ALD) technique [37].

The silica layers were grown employing three different precursors: (3-aminopropyl) triethoxysilane (H_2_N(CH_2_)_3_ Si(OCH_2_CH_3_)_3_) kept at a temperature of 100 °C; water (H_2_O) at 60 °C and ozone (O_3_). The reaction temperature in the chamber was fixed to 180 °C. Each layer was deposited in a total of 170 ALD cycles, which results in a thickness of around 10 nm, according to the estimated deposition rate of 0.06 nm/cycle [38].

The iron oxide (III) layer uses ferrocene (Fe(C_5_H_5_)_2_) as precursor along with ozone, with a reaction temperature in the chamber of 230 °C. The layer was deposited after a total of 1150 cycles, resulting in a thickness for the layer of 25 nm, according to the estimated deposition rate of 0.022 nm/cycle.

### 2.3. Fabrication of the Nanowires Core by Electrodeposition

The core of the nanowires, composed of an FeCo alloy, is grown by electrochemical deposition. Prior to the electrodeposition, a conductive gold layer, which will act as an anodic electrode during the process, is sputtered and electrodeposited from a commercial electrolyte (Orosene 999). The FeCo electrolyte is composed of 0.16 M (CoSO_4_) + 0.09 M (FeSO_4_) + 0.06 M (C_6_H_8_O_6_) + 0.16 M boric acid (H_3_BO_3_). The CoSO_4_, C_6_H_8_O_6_, and H_3_BO_3_ are mixed together and dissolved in 200 mL of H_2_O, heating the dissolution to 40 °C while bubbling it up with N_2_. The bubbling helps with the removal of oxygen present in the system, after which FeSO_4_ is added (it would oxidize easily otherwise). After the dissolution is completed, it is left to cool down before use. The electrodeposition has been performed at a potentiostatic voltage of −1.8V vs. Ag/AgCl reference electrode for 5 min.

In order to recrystallize hematite (Fe_2_O_3_) in the nanotubes into magnetite (Fe_3_O_4_) phase, a new batch of samples underwent a thermal reduction treatment procedure in a furnace by annealing at 350 °C in a controlled H_2_ (5%) + Ar (95%) atmosphere for 3 h [39,40]. The temperature of the thermal reduction treatment is lower enough to modify the structural and magnetic properties of the FeCo inner core of the core/shell heterostructure.

### 2.4. Characterization Techniques

Scanning electron microscopy (SEM, JEOL 5600, JEOL, Akishima, Tokyo, Japan) equipped with an energy dispersive X-ray microanalysis system (EDX, Inca Energy 200, Oxford Instruments, Abingdon, UK) was employed to perform a morphological and compositional characterization of the samples. Transmission electron microscopy (TEM, JEOL JEM 2100, JEOL, Akishima, Tokyo, Japan) suited with an EDX detector (X-MAX, Oxford Instruments, Abingdon, UK) and operating in scanning transmission mode was employed for the EDX linescan analysis of the layered core/shell nanowires hetero-structure.

The magnetic study is performed using a vibrating sample magnetometer (VSM-Versalab, Quantum Design, San Diego, CA, USA) under a magnetic field up to ±3 T and temperatures ranging from 50 K to 400 K. Furthermore, hysteresis loops of individual core/shell nanowires have been measured at room temperature (RT) by means of the magneto-optical Kerr effect using a NanoMOKE^®^3 from Durham Magneto Optics Ltd. (Cambridge, UK).

### 2.5. First Order Reversal Curve (FORC) Method

FORC methodology is a powerful tool for the study of magnetic properties of materials and it is used to complement the magnetostatic study of the samples that the hysteresis loops would offer by themselves. Although it is not a purely standard characterization technique, the FORC method is increasingly being applied to unravel complex magnetic behaviors. An introduction to this methodology is available in many scientific reports of articles [41,42], reviews [43,44,45,46], books [47], and references therein. Very briefly, the idea of this method consists in assuming that the global magnetic hysteresis loop of the sample is composed of different magnetic contributions coming from different sections, elements, or materials that form the sample. The magnetic switching of an individual element is not exclusively dependent on the applied magnetic field but on the magnetic state of its neighboring surroundings which modifies the effective magnetic field that affects it. In order to apply the FORC method, a specific measurement protocol must be followed. After bringing the sample to magnetic saturation, the applied magnetic field is reduced to a certain reversal field $H_{r}$, after which the magnetization is recorded along the way back to saturation. Repeating this measurement protocol for several reversal fields ($H_{r}$), evenly spaced between positive and negative saturation fields, the FORC distribution $\rho \left(H_{b},{\;H}_{r}\right)$ can be calculated as the mixed derivative of the magnetization (m) with respect to the reversal field, $H_{r}$ and the applied magnetic field along the way back to positive saturation, $H_{b}$, as shown in Equation (1). This distribution is frequently represented as a contour plot where one can detect a hysteretic process taking place in the $H_{r}$, $H_{b}$ space.

(1) $$\rho \left(H_{b},{\;H}_{r}\right)=\;-\frac{1}{2}\frac{\partial m}{\partial H_{b}\partial H_{r}}$$

## 3. Results and Discussion

### 3.1. Samples Fabrication

In order to obtain information regarding the intrinsic magnetic behavior of the different components (magnetic core and magnetic shell) and investigate the different magnetic interactions that may exist among them, different samples were synthesized and studied in this work. All the samples, together with their acronyms are summarized in Table 1. First of all, two HA-NAM templates were coated just with the SiO_2_/Fe_2_O_3_/SiO_2_ trilayer, corresponding then to those samples where only the nanotube shells are present. The as-deposited (AD) sample does not contain any magnetic phase whilst the sample thermally treated (TT) is formed of ferrimagnetic magnetite nanotubes. In two additional samples, a FeCo core nanowire is electrodeposited inside the NTs of SiO_2_/Fe_2_O_3_/SiO_2_ coated pores of the alumina membranes. In this case, the as-deposited sample is just the one where only the core is ferromagnetic. After following an appropriate thermal reduction treatment under a controlled atmosphere, both the core and the shell turn into magnetics, being the characterization of this bimagnetic sample the main objective of this study.

**Figure 2:**
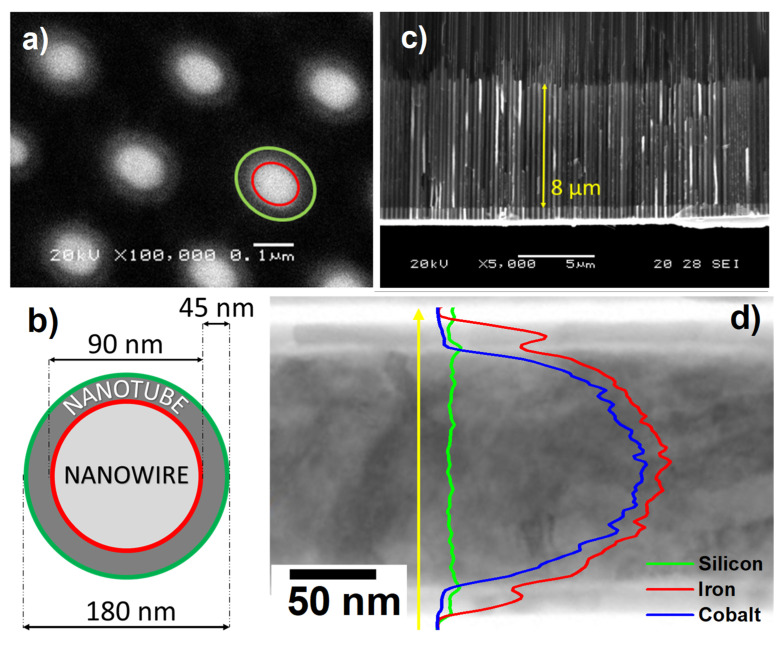
(**a**) Top-view scanning electron micrography of the nanowire/nanotube core/shell system. (**b**) Scheme of the dimensions of the core/shell elements grown in this work. (**c**) Cross-sectional view scanning electron micrography of the nanowire/nanotube system and the mean length of the elements. (**d**) STEM image and EDX linescan analysis performed of a single core/shell NW showing its layered heterostructure.

The geometry of the samples has been analyzed by SEM. Figure 2a displays a top view SEM image of the NWNT-AD array, where the different parts that compose the system are identified according to the scheme illustrated in Figure 2b, with an external diameter of the nanotubes shell of around 180 nm and an inner-core of FeCo nanowire with 90 nm in diameter. It is worth mentioning that the apparent elliptical cross-section displayed by the NWNT-AD core/shell nanowires in Figure 2a should be ascribed to artifacts during image acquisition at high magnifications in SEM. The cross-section of the NTNW embedded in the HA-NAM is shown in Figure 2c. The trilayered structure of the core/shell NWs can also be observed in the EDX linescan analysis performed by TEM on a single NW and shown in Figure 2d, where the peaks corresponding to the outer iron oxide shell, the SiO_2_ interspacing, and the metallic FeCo nucleus are clearly detected. After a deposition time of 5 min, the mean length of the nanowires is around 8 μm. An EDX analysis was performed on a test sample without the deposition of the Fe_2_O_3_ shell (it would false the nanowires composition otherwise), which confirmed that the average NWs composition was Fe_56_Co_44_.

### 3.2. Magnetic Properties of the Magnetite Nanotubes

**Figure 3:**
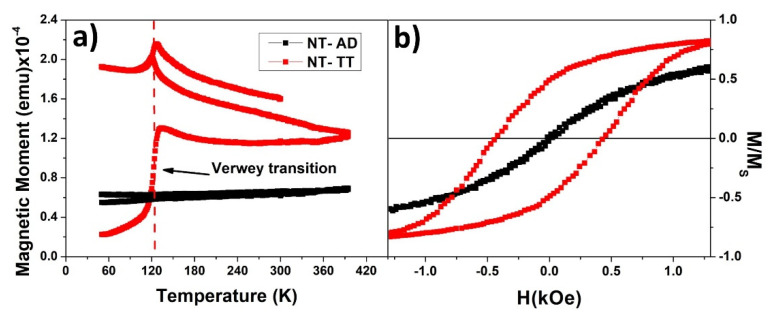
(**a**) ZFC-FC-FH magnetization curves for the shell of Fe_2_O_3_ NT-AD (black) and Fe_3_O_4_ NT-TT (red), respectively. (**b**) Normalized VSM hysteresis loops measured by applying the magnetic field along the parallel direction to the hematite (black) and magnetite (red) nanotubes length.

In order to confirm the magnetic phase transformation into magnetite of the hematite nanotubes after the thermal reduction treatment under a controlled atmosphere, a thermomagnetic, M(T), study has been performed. In Figure 3a, the Zero-Field-Cooling, Field Cooling, Field Heating (ZFC-FC-FH) protocol M(T) curves measured at a constant magnetic field of 100 Oe is displayed, unmistakably identifying the presence of magnetite phase in the NT shell system due to the detection of the Verwey transition appearing at 125 K [48,49].

To study the relevance of the shell magnetite phase in the nanotube system, we withdrew some samples from the fabrication process after the ALD procedure but before the nanowires were electrodeposited. It is important to note that for sake of clarity, due to the prevalence of the shape anisotropy for these kinds of systems, the magnetostatic characterization has been restricted to the particular case when applying the external magnetic field along the longitudinal axis of the core/shell nanostructure, which is, in fact, the easy magnetization axis of both, nanowires and nanotubes. A comparison of the magnetic hysteresis loops measured on the nanotube system, before (NT-AD) and after (NT-TT) undergoing the thermal treatment for the hematite phase transformation into magnetite, can be seen in Figure 3b. After performing the thermal treatment under a controlled atmosphere, the resulting shell of magnetite nanotubes has a higher coercivity value, increased from $H_{C}=24\;Oe$ for hematite NTs to $H_{C}=427\;Oe$ for magnetite NTs.

### 3.3. Study of the Core/Shell System

The main study of this work has been performed to compare the magnetic behaviour of three samples. We designated the samples in the following way: a first sample which only has the magnetic nanotubes (NT-TT), a second sample with magnetic nanowires but non-magnetic nanotubes (NWNT-AD), and a third sample that has both magnetic nanotubes and nanowires (NWNT-TT). The magnetic behavior of the nanotube and nanowire systems has been characterized individually through the study of the hysteresis loop measured along the axial direction for each sample and then, the corresponding hysteresis loop for the coupled bimagnetic core/shell system was also analyzed.

**Figure 4:**
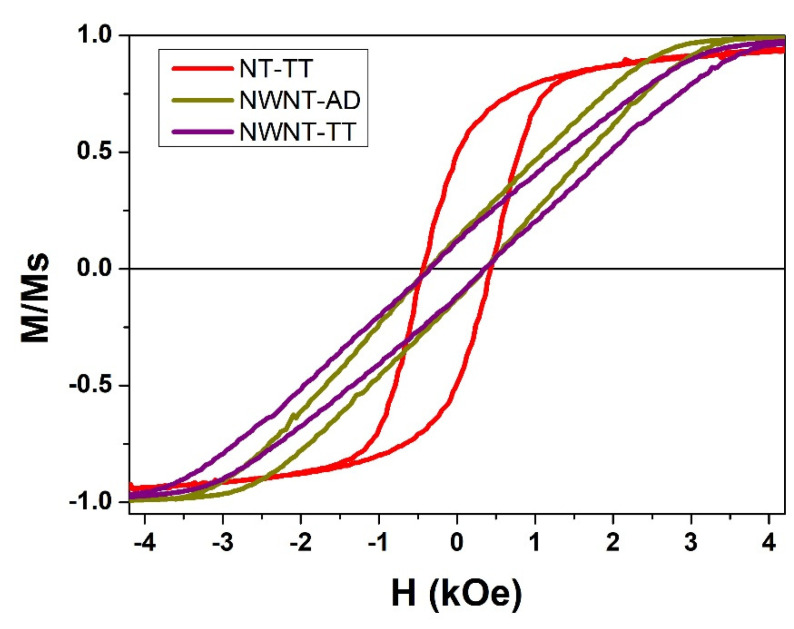
Comparison of the normalized hysteresis loops measured at 300 K for the NT-TT, NWNT-AD and NWNT-TT samples by applying the external field along the parallel direction with respect to the nanowire/nanotube longitudinal axis.

From the axial hysteresis loops plotted in Figure 4, we can extract a basic idea regarding the magnetic events that take place in each system [50]. By analyzing the slope of the histeretic curves, one can deduce that the NT-TT sample, which only contains the magnetite nanotubes, is a magnetic system where the individual elements which constitute the sample do not magnetically interact so much among them, as its hysteresis loop appears to be straight and relatively parallel to the vertical axis. However, for the NWNT-AD sample, which only contains the Fe_56_Co_44_ ferromagnetic nanowires, the loop displays a sharper slope than the NT-TT one. This fact indicates that the magnetic elements involved in the system are magnetostatically interacting with each other to a higher degree than in the previous sample. Therefore, the switching fields needed to reverse the magnetization of the magnetic elements of the NWNT-AD sample are altered, making the switching happen at a lower or higher field value (depending on each individual element) than those required to switch them when they are isolated by separately. Lastly, the NWNT-TT sample, which is composed of the core/shell bimagnetic nanostructures, shows an even gentler and steeper slope than the nanowires sample (NWNT-AD), indicating that the two magnetic elements of the core/shell sample turn into even much more interacting between them after the magnetite nanotubes have been added to the system. The individual magnetic elements of the sample are switching their magnetization state at even lower or higher external field values due to the more intense neighboring magnetic field.

**Figure 5:**
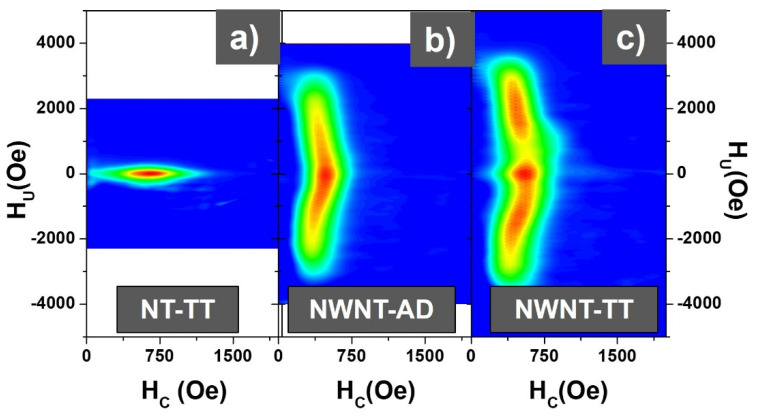
FORC distributions for (**a**) NT-TT sample with only magnetite nanotubes; (**b**) NWNT-AD sample with only FeCo ferromagnetic nanowires; and (**c**) NWNT-TT bimagnetic core/shell sample.

In order to further expand this concept, we rely on the more complex FORC methodology by obtaining the FORC distributions of the samples at 300 K, all of them measured by applying the magnetic field parallel to the nanowires/nanotubes long axis. The FORC distributions for all three samples are collected in Figure 5. Figure 5 (left panel) represents the FORC distribution corresponding to the NT-TT sample which denotes a single distribution along the coercivity axis, H_c_, thus indicating a weakly interacting system as it was also inferred from the corresponding hysteresis loop, where the wide FORC distribution indicates the broad switching field values of each NT. In contrast to the previous one, the FORC distribution for the NWNT-AD sample, shown in Figure 5 (middle panel), displays an elongated distribution along the interaction axis, H_u_, which indicates a strongly interacting system where each FeCo nanowire influences the magnetic state of its neighbors. Finally, in Figure 5 (right panel) the NWNT-TT FORC distribution displays a quite similar elongated distribution along the H_u_ axis to the NWNT-AD, but there are visible differences in the shape of the distribution. Although apparently, it could resemble the linear superposition of the two previous FORC distributions, i.e., direct superposition of contributions from NWs and NTs, a deeper look into it suggests otherwise. Instead of a FORC diagram comprising the superposition of two independent FORC distributions, it looks that the central distribution along H_u_ = 0, which is in a first instance ascribed to the magnetite nanotubes, shifts the whole magnetization reversal processes of the FeCo nanowires to higher and lower values of the interaction field. This could be explained by considering that the magnetization reversal of the nanotubes, which occurs completely at a certain value of the applied field, introduces a jump or an offset in the effective magnetic field that affects the whole sample and thus to the nanowires.

**Figure 6:**
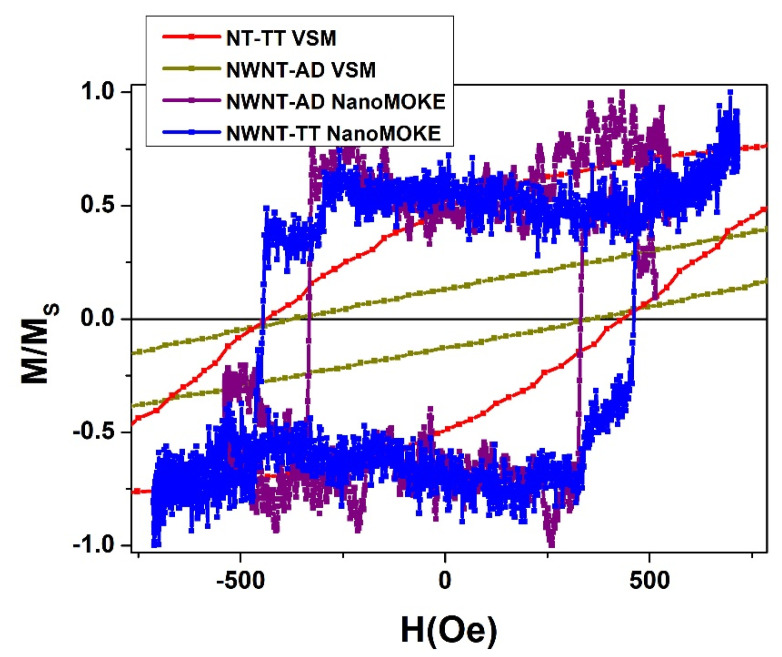
VSM hysteresis loops of the NT-TT (red) and NWNT-AD (dark yellow) arrays. MOKE hysteresis loops of individual Fe_56_Co_44_ nanowires (purple) and core/shell bimagnetic nanowire (blue). All hysteresis loops are measured by applying the external field along the parallel direction with respect to the nanowires/nanotubes longitudinal axis.

Previous analyses point out that the difference between the nanowire and core/shell systems is the delay of the switching process of the nanowires, represented by a shift towards higher values of the external magnetic field. The presence of the magnetic nanotubes in the core/shell nanowire system, increased the magnetostatic interaction felt by the nanowires, so that the antiparallel coupling between the core and shell is favored. However, magnetostatic interaction among neighboring nanowires is still dominant, as inferred from hysteresis loops and also from FORC analyses. In order to better clarify the magnetization reversal process of individual core/shell nanowires, magneto-optical Kerr effect measurements have been performed with the applied magnetic field parallel to the core/shell longitudinal axis. In Figure 6, the VSM and MOKE parallel hysteresis loops are compared for several samples. The NT-TT VSM and NWNT-AD VSM parallel loops are included as a reference for the magnetization reversal of the nanotubes and the nanowires, respectively. As it can be appreciated from the comparison between the respective VSM and MOKE measurements, the switching field of the single nanowire (NWNT-AD sample with no magnetic shell measured by MOKE, in purple color) matches well with the coercive field value of the FeCo nanowires array measured by VSM (dark yellow color). Furthermore, the blue-colored loop in Figure 6, corresponding with the MOKE hysteresis loop of a single core/shell bimagnetic nanostructure, presents two well-differentiated magnetization jumps. The first one seems to correspond to the ferromagnetic FeCo nanowire in the core, as compared with the one observed for the NWNT-AD sample (purple and dark yellow colors), whilst the second reversal of magnetization is ascribed to the shell of magnetite nanotube, as it is located near the coercive field value of the NT-TT sample obtained from the corresponding VSM hysteresis loop (red color).

## 4. Conclusions

A cylindrical coaxial bimagnetic core/shell structure composed of Fe_56_Co_44_ nanowires and SiO_2_/Fe_2_O_3_/SiO_2_ multilayered nanotubes were successfully fabricated and magnetically characterized. Aiming to design a more complex magnetostatic system, some samples containing SiO_2_/Fe_2_O_3_/SiO_2_ nanotubes underwent further thermal treatment in an H_2_-controlled atmosphere (5% H_2_ + 95% Ar) to crystallize the hematite (Fe_2_O_3_) phase into magnetite (Fe_3_O_4_) one. From the comparison between the hysteresis loops of the nanotube, nanowire, and nanowire/nanotube systems it was found that the nanotube system does not display a highly magnetic interactive behavior while the nanowire system displays higher magnetostatic dipolar interaction between its elements. Additionally, the bimagnetic nanowire/nanotube system showed an even higher degree level of magnetostatic interaction among their constituting elements. However, in order to fully understand the different magnetization processes occurring in the samples, the First-Order Reversal Curves methodology was introduced. The FORC analysis performed on the shell nanotube samples showed a weakly magnetostatic interaction system, whereas the nanowire and nanowire/nanotube samples’ magnetic behavior was dictated by a system with strongly magnetostatic interacting elements. A deeper analysis of the core/shell nanowire/nanotube sample shows evidence of the magnetostatic coupling of dipolar origin between the two magnetic parts, the core, and the shell, of the bimagnetic nanostructure, in such a way that their magnetic interactions cause the delay in the switching of their magnetic state as compared to their individual magnetic behaviors separately. Finally, the two-step switching of the magnetization reversal process for the bimagnetic nanowire/nanotube system was confirmed by MOKE measurements performed on several individual elements of the core/shell sample.

## Figures and Tables

**Table 1:** Designations used for the different samples’ terminology employed during this work.

Acronym	Nanotubes (Shell)	Nanowire (Core)	Thermal Treatment	Magnetic	
				Shell	Core
NT-AD	SiO_2_/Fe_2_O_3_/SiO_2_	None	No	×	×
NT-TT	SiO_2_/Fe_3_O_4_/SiO_2_	None	Yes (5% H_2_ + 95% Ar)	√	×
NWNT-AD	SiO_2_/Fe_2_O_3_/SiO_2_	FeCo	No	×	√
NWNT-TT	SiO_2_/Fe_3_O_4_/SiO_2_	FeCo	Yes (5% H_2_ + 95% Ar)	√	√
